# Whole-Genome Sequence of *Bacillus cereus* AR156, a Potential Biocontrol Agent with High Soilborne Disease Biocontrol Efficacy and Plant Growth Promotion

**DOI:** 10.1128/genomeA.00886-17

**Published:** 2017-08-31

**Authors:** Chun-Hao Jiang, Yun Chen, Fang Yan, Zhi-Hang Fan, Jian-Hua Guo

**Affiliations:** a Department of Plant Pathology, College of Plant Protection, Nanjing Agricultural University, Key Laboratory of Monitoring and Management of Crop Diseases and Pest Insects, Ministry of Agriculture, Engineering Center of Bioresource Pesticide in Jiangsu Province, Nanjing, China; b College of Life Sciences, State Key Laboratory of Crop Genetics and Germplasm Enhancement, Nanjing Agricultural University, Nanjing, China; c Institute of Biotechnology, Zhejiang University, Hangzhou, China

## Abstract

*Bacillus cereus* AR156 was originally isolated from the forest soil of Zhenjiang, a city in China. To shed new light on the molecular mechanisms underlying the biological control of soilborne pathogens, the whole genome of this strain was sequenced. Here, we report the draft genome sequence of this strain, consisting of a single circularized contig measuring 5.66 Mb, with an average GC content of 35.5% and 5,367 open reading frames.

## GENOME ANNOUNCEMENT

*Bacillus cereus* AR156 is a plant growth-promoting rhizobacterium, originally isolated from a garden tree soil rhizosphere in Nanjing, China, that could protect tomato plants against bacterial wilt caused by *Ralstonia solanacearum* and the root knot nematode *Meloidogyne incognita*. We previously found that *B. cereus* AR156 can induce systemic resistance to *Pseudomonas syringae* pv. tomato DC3000 by simultaneously activating the SA and JA/ET signaling pathways via an NPR1-dependent mechanism (1–3). The biocontrol mechanisms of *B. cereus* AR156 are currently unclear. To better understand the biocontrol and plant growth-promoting mechanisms of *B. cereus* AR156, the whole genome of the strain was determined by 454 sequencing. Total DNA was prepared as described in the GS FLX Titanium general library preparation kit (454 sequencing) and then sequenced according to the GS FLX Titanium sequencing method manual. The 239,826 generated reads were assembled into contigs using the GS *de novo* assembler. The genome coverage, which was based on the sequenced DNA, was found to be approximately 15.3-fold. Primer walking and PCR-based techniques were applied to close the remaining gaps. All of the manual editing steps were performed using the Consed software package (4, 5). Prediction of the operons in the *B. cereus* AR156 chromosomes was performed with an algorithm that combines intergenic distance and phylogenetic information (6), and tRNAs and transfer-messenger RNAs (tmRNAs) were predicted using the ARAGORN program (7); rRNA was obtained by using RNAmmer version 1.2 (8).

The 5.66-Mb *B. cereus* AR156 genome was composed of four replicons, a circular chromosome that encodes 5,367 open reading frames (ORFs) and three plasmids, pAR460, pAR41, and pAR10. Among the 5,367 ORFs, 3,333 clusters of orthologous groups were identified. Riboswitches play a very important role in a number of cellular processes, such as regulation of gene expression, tRNA processing, and protein secretion (9). The riboswitches in the *B. cereus* AR156 genome sequence were predicted with a method using pHMM. By applying default parameters, 36 riboswitches were identified in the *B. cereus* AR156 genome (eight binding *S*-adenosylmethionine, six specific for thiamine pyrophosphate, eight for purines, eight for PreQ, two for flavin mononucleotide, two for lysine, and one each for Glms and Cob). *B. cereus* AR156 displays a robust swarming phenotype. In *B. cereus* AR156, there are four genes whose products are weak and similar to those of *swr* in *B. subtilis*. It has been reported that the hook-associated flagellar proteins HAP1 and HAP2 are affected differently by exudates secreted by plant roots (10). There are four hook-associated flagellar proteins identified in *B. cereus* AR156. In addition to the *B. cereus* AR156 chromosome, its pAR460 and pAR41 plasmids also contain peptide antibiotics; pAR460 encodes four other bacteriocins. The pAR460 plasmid also encodes one camelysin and one microbial collagenase. Several genes similar to linear gramicidin synthetase subunits and linear gramicidin dehydrogenase LgrE were found in pAR460. Linear gramicidin is a pentadecapeptide antibiotic that forms a membrane channel (11, 12). The other plasmid, pAR41, also encodes four lantibiotic acids as modification enzymes and six bacteriocins.

### Accession number(s).

The complete genome sequence for *B. cereus* AR156 reported here has been deposited in GenBank under the accession numbers CP015589 (chromosome), CP015590 (plasmid pAR10), CP015591 (plasmid pAR41), and CP015592 (plasmid pAR460).
